# Clinic Reports

**Published:** 1880-12

**Authors:** L. D. Wilson


### CLINIC REPORTS.

*University of Michigan Medical Department. Surgical Clinic of Prof. D. Maclean, Sep't 20, 1880. Reported by L. D. Wilson*

Malignant tumor—Epulis. The patient was a man aged thirty-five years. The tumor was on the right side of the lower jaw, covering some of the teeth and filling the one side of the mouth. There was a fistulous opening in the lower part of the cheek which communicated with the tumor. The tumor also involved a portion of the cheek tissues. The patient being anæsthetized, an incision was made along the lower border of the jaw and up through the lower lip. It was then disarticulated on the right side and cut through at the symphasis with a saw. The operation occupied about an hour and a quarter.

OCT. 13TH.—EPULIS TUMOR--MALIGNANT.

This patient was a lady fifty years of age. The seat of this tumor was in the upper maxilla of the right side, covering the two bicuspids. Fourteen years ago the patient had a molar tooth extracted, but two roots were left in the jaw, and this was the supposed origin of the tumor. The tumor was growing fast.

The patient being chloroformed, Prof. Maclean made an incision, first through the upper lip, curving around the right side of the nose, and thence horizontally outwards to about half way between the nose and ear. The soft tissues were dissected back, and the superior maxilla sawed through below the floor of the orbit, and all below it removed. The patient was present at the clinic eight days after the operation, with the parts healing nicely.

NOV. 13TH, '80—EPITHELIOMA.

The patient was a man aged thirty-eight years. He had been in the habit of smoking a short pipe ; and his epithelioma was probably the result of that pernicious habit.

In the highlands of Scotland where the pipe is used much by the shepherds, epithelioma of the lip is very common, and is invariably found on the side on which the pipe was held. In this case the disease was in the center of the lower lip, and not having yet extended far, a V shaped piece was cut away without the ad ministration of an anæsthetic. The cut edges were held in apposition by the figure of eight sutures; as in hare lip.

NOV. 13TH.—POLYPUS OF THE NOSE.

Man aged forty-nine. It was removed with forceps in pieces, passed back in the posterior nares. No anæsthetic was given and not much hemorrhage occurred. There is not as much pain as would be supposed in their removal.

If hemorrhage occurs the proper method of stopping it is to pass a rubber catheter through the nostril into the mouth, tie to it a string, to which is fastened a tampon, and draw it tightly into the posterior nares.

NOV. 16TH.—HARE LIP.

This patient was a child one and one-half years of age, and in good health The hare lip was double, and a double cleft palate also existed. The child was chloroformed, and the operation begun. In the alveolar process between the two clefts were the central incisor teeth, which were allowed to remain. The section of lip in the center was closely adherent to the process, as were also the side portions. These were all dissected up, and instead of cutting away entirely the middle section (which is often done in cases of double hare lip) it was accurately fitted in.

The hare lip needle, silver sutures and horse hair sutures were used and the parts bound firmly together. Formerly hairs were taken from the tails of horses and used in that condition for sutures; but they are now prepared for the purpose, and are in the market.

When the operation was completed a compress was put on the child's cheeks. It consisted of a band of about four-fifths of a circle, containing a steel spring, and this to be put around the neck of the child, so that the ends would press upon and hold the cheeks in place, to prevent movement of the parts when the child would cough, struggle, etc. This band was supported by two straps that buckled over the head, and a third one ascending from the back and fastened to the others above.

Although the operation for the correction of hare lip is a comparatively simple one, it can not be hurried through with, but must be deliberately and carefully performed. If it is not, disappointment will be the result.

